# Can Deep Learning-Based Volumetric Analysis Predict Oxygen Demand Increase in Patients with COVID-19 Pneumonia?

**DOI:** 10.3390/medicina57111148

**Published:** 2021-10-22

**Authors:** Marie Takahashi, Tomoyuki Fujioka, Toshihiro Horii, Koichiro Kimura, Mizuki Kimura, Yurika Hashimoto, Yoshio Kitazume, Mitsuhiro Kishino, Ukihide Tateishi

**Affiliations:** 1 Department of Diagnostic Radiology, Tokyo Medical and Dental University Hospital of Medicine, Tokyo 113-8510, Japan; tkhsdrnm@tmd.ac.jp (M.T.); horii.m66.tmdu@gmail.com (T.H.); kmrdrnm@tmd.ac.jp (K.K.); 150421ms@tmd.ac.jp (M.K.); h_yurika_7@yahoo.co.jp (Y.H.); ktzmmrad@tmd.ac.jp (Y.K.); ksnmrad@tmd.ac.jp (M.K.); ttisdrnm@tmd.ac.jp (U.T.); 2 Trauma and Acute Critical Care Medical Center, Tokyo Medical and Dental University Hospital of Medicine, Tokyo 113-8510, Japan

**Keywords:** chest imaging, COVID-19, deep learning, radiology, chest CT, oxygen demand, deterioration, prediction

## Abstract

*Background and Objectives*: This study aimed to investigate whether predictive indicators for the deterioration of respiratory status can be derived from the deep learning data analysis of initial chest computed tomography (CT) scans of patients with coronavirus disease 2019 (COVID-19). *Materials and Methods*: Out of 117 CT scans of 75 patients with COVID-19 admitted to our hospital between April and June 2020, we retrospectively analyzed 79 CT scans that had a definite time of onset and were performed prior to any medication intervention. Patients were grouped according to the presence or absence of increased oxygen demand after CT scan. Quantitative volume data of lung opacity were measured automatically using a deep learning-based image analysis system. The sensitivity, specificity, and area under the receiver operating characteristic curve (AUC) of the opacity volume data were calculated to evaluate the accuracy of the system in predicting the deterioration of respiratory status. *Results*: All 79 CT scans were included (median age, 62 years (interquartile range, 46–77 years); 56 (70.9%) were male. The volume of opacity was significantly higher for the increased oxygen demand group than for the nonincreased oxygen demand group (585.3 vs. 132.8 mL, *p* < 0.001). The sensitivity, specificity, and AUC were 76.5%, 68.2%, and 0.737, respectively, in the prediction of increased oxygen demand. *Conclusion:* Deep learning-based quantitative analysis of the affected lung volume in the initial CT scans of patients with COVID-19 can predict the deterioration of respiratory status to improve treatment and resource management.

## 1. Introduction

The novel coronavirus disease 2019 (COVID-19) has rapidly spread worldwide since it was first reported in Wuhan, Hubei Province, China, in December 2019. Worldwide, more than 226,800,000 people have been infected by 17 September 2021, and more than 571,000 people have been affected daily. In Japan, 1,663,024 people have been infected, of which 17,030 have died [1,2].

While many patients with COVID-19 are asymptomatic or present with mild symptoms and do not require hospitalization, the increasing number of patients with moderate-to-severe respiratory conditions (requiring oxygenation or mechanical ventilation) depletes medical and human resources as well as disrupts daily practices [3]. There are only few facilities that have the capacity to treat a high number of patients with severe COVID-19 who require mechanical ventilation or intensive care. Therefore, it is important to assess the risk of exacerbation in individual cases to determine the optimal distribution of patients and allocation of medical and human resources [4]. Older age, obesity, chronic obstructive pulmonary disease (COPD), serious heart disease, and several other comorbidities exacerbate the medical risks. Unfortunately, imagining techniques for predicting such exacerbations for individual cases have not yet been developed [5,6].

Computed tomography (CT) is a sensitive imaging modality for the detection of pneumonia. Nonsegmented pure ground-glass opacities (GGOs) in the peripheral side of the lung are typical in the images of early-stage COVID-19 pneumonia. As the disease progresses, the number of lesions increases, and a crazy-paving pattern can be observed in the GGOs. GGOs become mixed with consolidation, and consolidation increases as the condition worsens. At the time of healing, consolidation decreases and becomes faint with a cord-like structure [7,8,9]. Knowledge of these typical processes and the resulting imaging patterns may be used as a tool for estimating the approximate stage of an individual patient based on CT findings. The area of the lesion usually shrinks during the healing phase and expands as it becomes more severe [10,11]. Therefore, we examined whether the area of the GGOs or consolidation could serve as a predictive indicator of the severity of pneumonia. In addition, CT findings are known to dynamically change over time, and previous studies revealed that they peak approximately 9–13 days after onset [10] (6–11 days in a study by Wang et al. [8]). We thought that stratification of the time from onset would increase the accuracy of predicting aggravation.

In recent years, artificial intelligence (AI), particularly deep learning, has been significantly developed and has been applied to medical image classification, object detection, semantic segmentation, etc. [12,13,14,15,16]. Moreover, it has been reported that the use of AI technology can greatly improve the accuracy of tasks that are difficult and time-consuming for humans to perform alone and, thus, save reading time. Several studies have been conducted on the detection, region extraction, and classification of COVID-19 pneumonia lesions on CT images, and the clinical usefulness of these studies have been verified [17,18,19,20,21].

Therefore, this study aimed to quantitatively analyze the chest CT scans of patients with COVID-19 using a deep learning-based system to investigate the association between image data and the deterioration of the patient’s respiratory status.

## 2. Materials and Methods

### 2.1. Study Population

**Figure 1:**
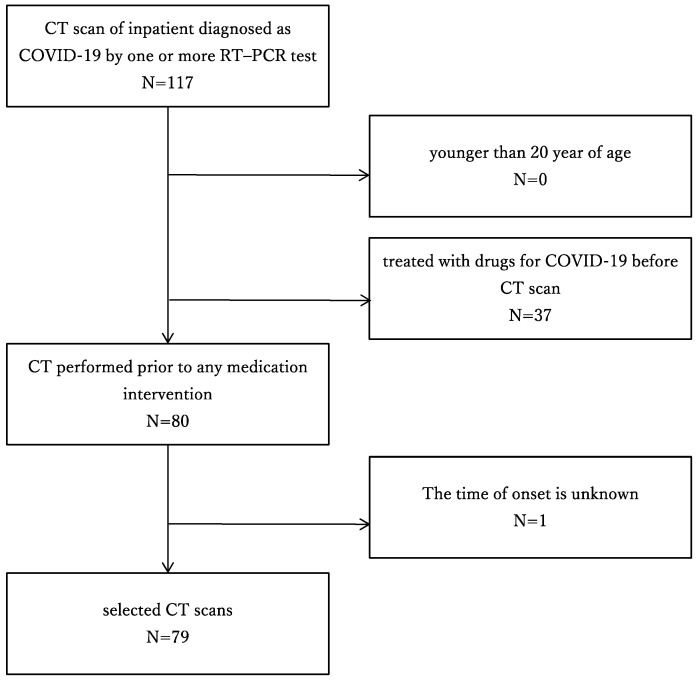
Flow chart of patient selection. CT: computed tomography; COVID-19: coronavirus disease 2019; RT-PCR: reverse transcription–polymerase chain reaction.

The medical ethics committee of our hospital approved this retrospective study and waived the requirement for written informed consent. In the present study, the inclusion criteria for patient enrollment were as follows: (a) Inpatient diagnosed as positive for COVID-19 by one or more reverse transcription polymerase chain reaction (RT-PCR) test and (b) underwent chest CT from April to June 2020. The exclusion criteria were as follows: (a) younger than 20 years of age, (b) treated with drugs for COVID-19 before CT scan, and (c) the onset was unclear (Figure 1).

After reviewing the database of radiology reports and clinical records at our institute, two board-certified radiologists with 6 and 10 years of imaging experience and a medical student extracted chest CT images and clinical data, including sex, age, symptoms, time of onset, time of CT scan, comorbidities (e.g., chronic kidney disease, COPD, obesity, serious heart disease, and diabetes), blood tests (white blood cell count [WBC], lymphocyte count % [LYM%], C-reactive protein [CRP], and lactate dehydrogenase [LDH]), and treatment (oxygen administration and mechanical ventilation). The radiologist has extracted the cases to be registered and the students were mainly in charge of entering the specified data into the sheet. In total, 79 CT scans of patients with COVID-19 were included in this study.

### 2.2. Chest CT Imaging

CT scan was performed based on the clinical judgment of the attending physician and was performed in the supine position, and the image was taken in the craniocaudal direction. In total, 47 of the CT examinations were conducted at our institution, and SOMATOM Edge Plus (Siemens Healthcare GmbH, Erlangen, Germany) with 64-detector rows was utilized. Conversely, 32 CT examinations were conducted outside our institutions, and several CTs with 4- to 320-detector rows were utilized. The acquisition parameters at our hospital were as follows: 120-kV tube voltage with automatic tube current modulation (150 mAs); tube rotation time, 0.28 s; beam collimation, 128 ch × 0.6 mm; and beam pitch, 1.5. By default, 2.0 mm chest CT images without interslice gap were reconstructed using a sharp tissue kernel (Bl57) and the filtered back-projection technique. Outside institutions, the slice thickness of the reconstructed images ranged from 1.25 to 5 mm.

### 2.3. COVID-19 Pneumonia Analysis Using Deep Learning System

**Figure 2:**
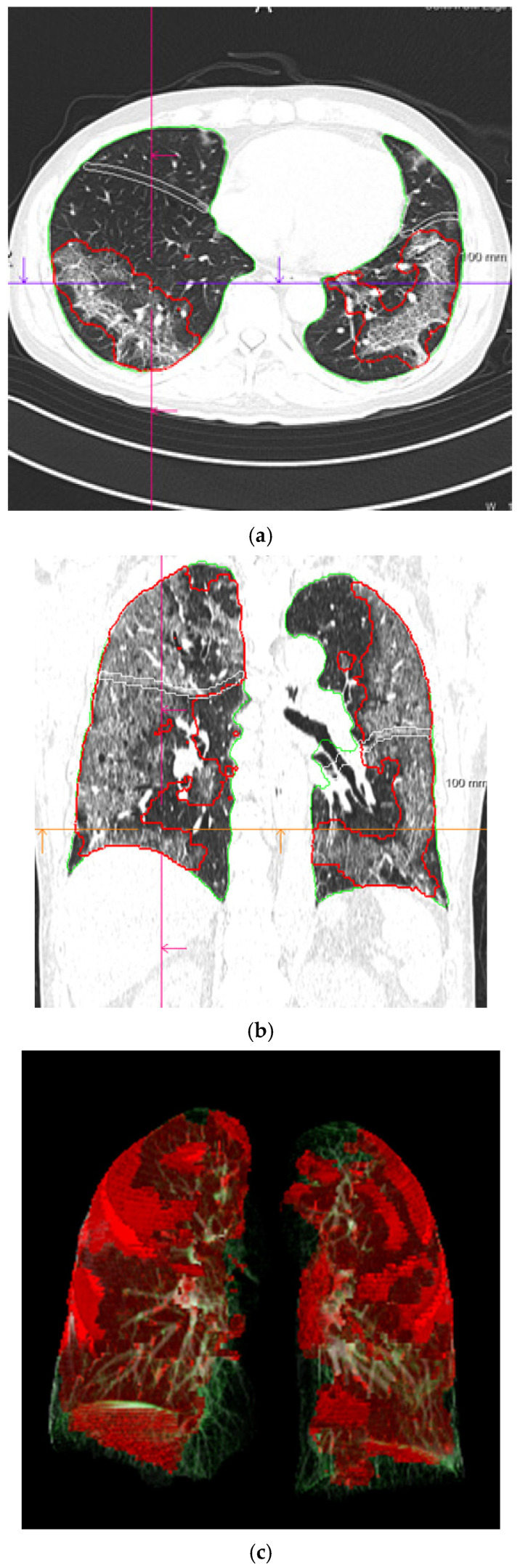
Lung and pneumonia area segmentation on CT images using deep learning-based analysis. Lung and pneumonia area on CT image segmented as three-dimensional data. A red border surrounds the pneumonia areas in the axial and coronal images (**a**,**b**). The pneumonia areas are visualized in a three-dimensional manner in red (**c**). CT: computed tomography.

The deep learning-based pneumonia analysis system (CT Pneumonia Analysis prototype, Siemens Healthcare GmbH, Erlangen, Germany) was used to quantitatively analyze the area of COVID-19 pneumonia on chest CT images. The system has been trained and tested using a dataset of 9749 three-dimensional chest CT volumes to automatically perform three-dimensional segmentation and quantification of the anomalous CT pattern GGOs (low opacities) and consolidations (high opacities) that are commonly present in COVID-19. Figure 2 presents an example of a segmented lung and pneumonia region on a CT image. The total opacity volume (mL), low opacity volume (mL), and high opacity volume (mL) were obtained.

### 2.4. Statistical Analysis

The statistical analyses in this study were conducted using EZR version 1.31 (Saitama Medical Center, Jichi Medical University, Saitama, Japan) [22] and IBM SPSS Stastics version 24 (International Business Machines Corporation, Armonk, NY, USA).

Descriptive statistics were used to express categorical variables as counts and percentages, and numeric or ordered variables were expressed as medians and 25th–75th percentiles. The variables were selected based on a clinical perspective. We compared the deterioration of the patient’s respiratory status and clinical and radiological factors (e.g., age, WBC, LYM%, CRP, and LDH); the continuous variables were compared using the Mann–Whitney *U* test, and the categorical variables were compared using the *χ*^2^ test.

To investigate the volume of pneumonia (total opacity, low opacity, and high opacity) as measured by the deep learning system and its relationship with oxygen demand and the deterioration of the patients’ respiratory status, a receiver operating characteristic (ROC) analysis was conducted. We calculated the sensitivity, specificity, and area under the ROC curve (AUC) to predict the deterioration of the patient’s respiratory status. An optimal cut-off value that was closest to the upper left corner was derived (the cut-off value with the highest sum of sensitivity and specificity). In each case, a *p*-value of < 0.05 was considered statistically significant.

Moreover, we divided the cases into three subgroups according to the time from onset to CT scan, early period (0–5 days), middle period (6–10 days), and late period (≥11 days), and analyzed each group separately. The day of onset was defined as the day when symptoms, such as fever, malaise, and respiratory symptoms, appeared.

Logistic regression analysis was performed with the dependent variable being the presence or absence of an increase in oxygen demand and the independent variables being volume of high opacity, low opacity, and overall opacity. Calibration was examined by the Hosmer–Lemeshow goodness-of-fit test (a non-significant test indicates good calibration) and by graphically examining the deviation between mean observed and mean predicted probabilities for increased oxygen in 10 equally sized groups of predicted risks.

## 3. Results

### 3.1. Patient Characteristics

Patients’ demographic data are presented in Table 1. The median age was 62 years, and 56 (70.9%) patients were male. The median duration between onset and the CT scan was 9 days [6,13].

Oxygen demand increased after the CT scan in 45 cases (57.0%) (increased oxygen demand group). The median age was higher for the increased oxygen demand group (65 years [54, 77] for the increased oxygen demand group and 51.5 years [36, 71.5] for the nonincreased oxygen demand group). In the increased oxygen demand group, 26 (57.8%) patients did not require oxygen at the time of the CT scan; however, oxygen demand increased afterward. In the nonincreased oxygen demand group, 30 (88.2%) patients never required oxygenation, and the remaining four (11.2%) patients required a maximum of 3 L/min of oxygen at the time of the CT scan, but the demand did not increase thereafter. In the increased oxygen demand group, 19 (42.2%) patients required mechanical ventilation during treatment, and extracorporeal membrane oxygenation was introduced for four (8.9%) patients.

### 3.2. Volume of Opacities as Predictive Factors

The opacity volume was significantly higher in the increased oxygen demand group than in the nonincreased oxygen demand group (585.3 mL vs. 132.8 mL, *p* < 0.001), as were the volume of high opacity (71.4 mL vs. 25.1 mL, *p* = 0.006) and the volume of low opacity (440.3 mL vs. 95.1 mL, *p* = 0.001).

**Figure 3:**
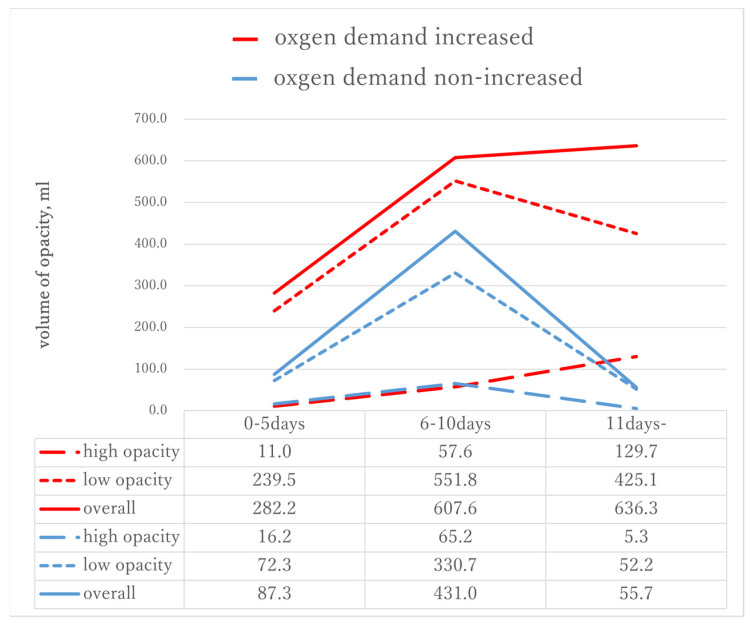
Change in the volume of each type of opacity on the chest CT from the time of the initial onset of symptoms. The low opacity volume peaked during the middle period and decreased during the late period for both the increased oxygen demand and the nonincreasing oxygen demand groups. Conversely, the high opacity volume increased during the late period for the increased oxygen demand group but decreased for the nonincreased oxygen demand group.

Table 2 and Figure 3 present the time course for the opacity volume of each type on chest CT from the onset of symptoms. The numbers of cases in each period were 17, 35, and 27, respectively. In addition, we compared the increased oxygen demand and nonincreased oxygen demand groups at each period. No significant difference was observed between the two groups in any type of opacity during the early period. The low opacity volume peaked in the middle period and decreased in the late period for both the increased oxygen demand and the nonincreased oxygen demand groups. Conversely, the high opacity volume increased during the late period for the increased oxygen demand group but decreased for the nonincreased oxygen demand group. Significant differences were observed in the low opacity volume during the middle period (551.80 mL vs. 330.69 mL, *p* = 0.044) and the high opacity volume in the late period (129.74 mL vs. 5.28 mL, *p* = 0.018).

**Figure 4:**
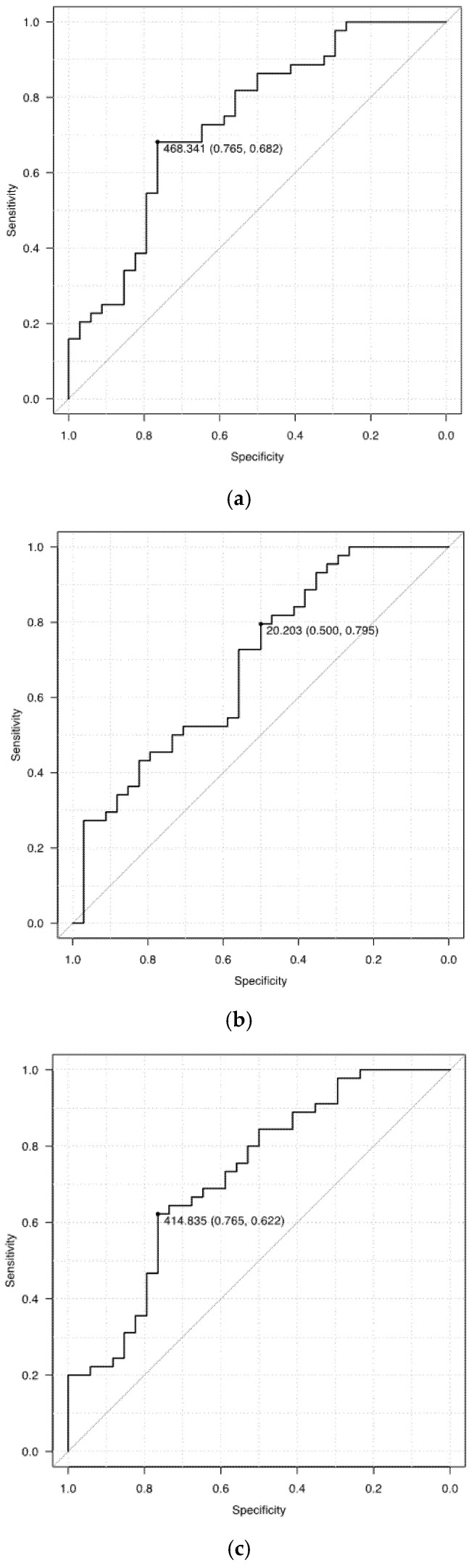
The receiver operating characteristic curves of the opacity volume as a predictor of oxygen demand. The sensitivity, specificity, and AUC of the volume of opacity were 76.5%, 68.2%, and 0.737 in predicting increased oxygen demand (**a**); 50.0%, 79.5%, and 0.691 for high opacity (**b**); and 76.5%, 62.2%, and 0.722 for low opacity (**c**), respectively.

Figure 4 presents the ROC curves of the opacity volume as a predictor of oxygen demand. The AUC was also estimated. The sensitivity, specificity, and AUC of the volume of opacity were 76.5%, 68.2%, and 0.737 (95% confidence interval [CI], 0.624–0.851) in predicting the increased oxygen demand; 50.0%, 79.5%, and 0.691 (95% CI, 0.572–0.811) for high opacity; and 76.5%, 62.2%, and 0.722 (95% CI, 0.607–0.837) for low opacity, respectively.

Table 3 describes the AUC for each type of opacity volume for increased oxygen demand in each period. The AUC values were low in all groups during the early period (0.45–0.65). The low opacity volume demonstrated a relatively high AUC value, but the high opacity volume showed a better value during the late period. The AUC value before the middle period of the high opacity volume did not reach 0.5, which was very low.

**Figure 5:**
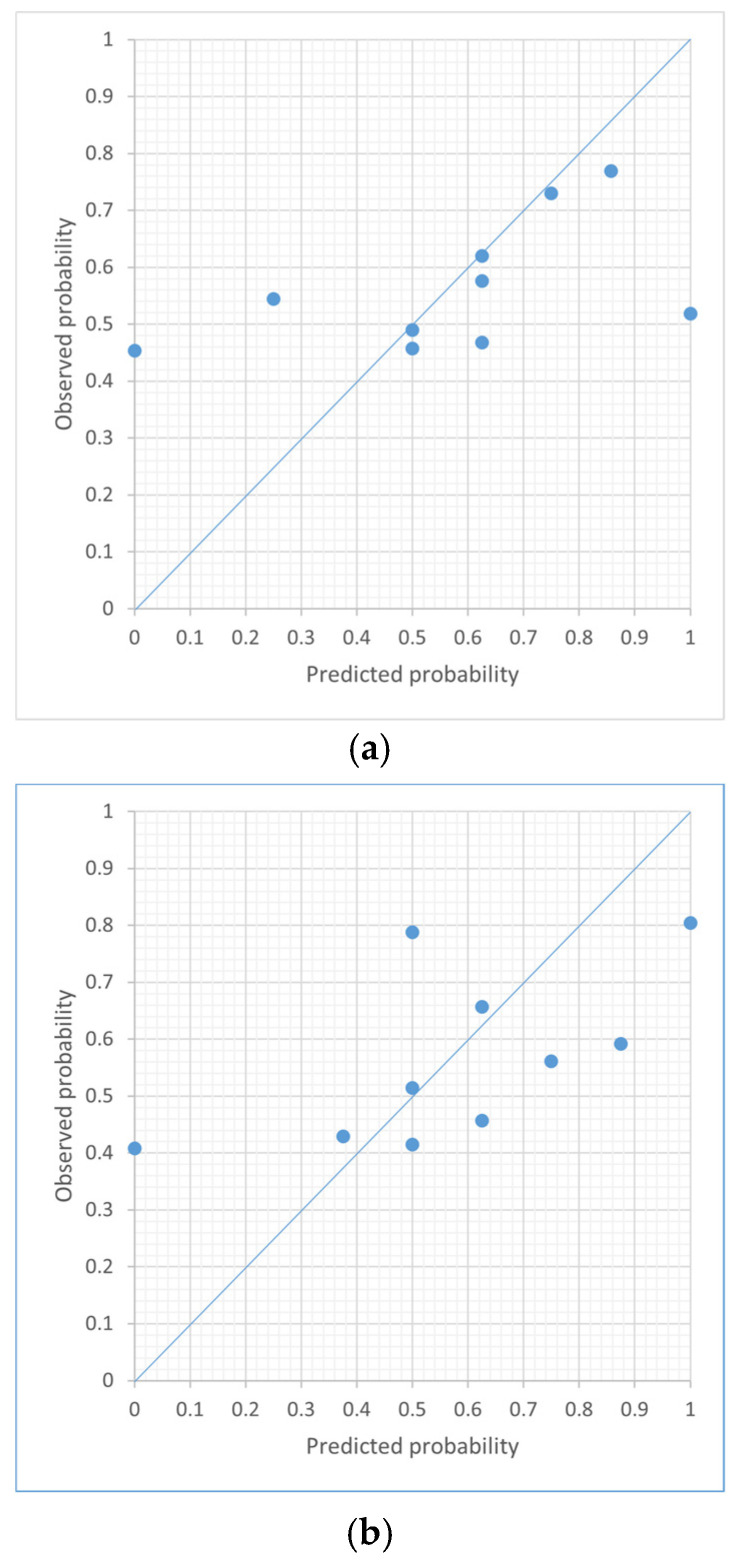

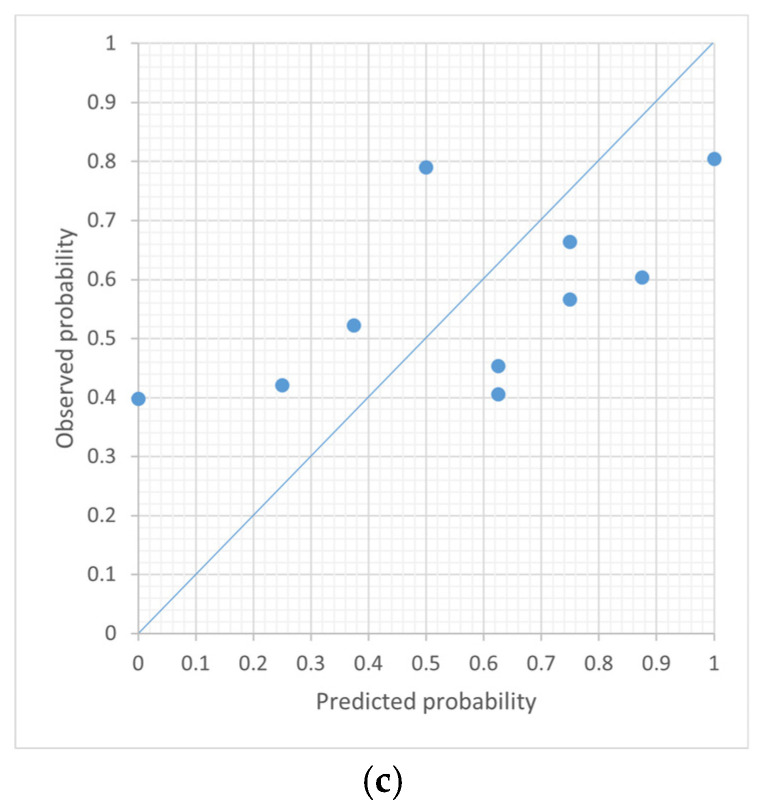
The calibration plots for volume of high, low, and overall opacity. The Hosmer–Lemeshow goodness of fit between the measured and predicted values showed that volume of low opacity (*p* = 0.056) (**b**) was higher than volume of high opacity (*p* = 0.022) (**a**) and overall opacity (*p* = 0.021) (**c**).

Figure 5 shows the calibration plots for volume of high, low, and overall opacity, respectively. Volume of high opacity and overall opacity are poorly calibrated because they have some groups with over- or under-predicted risk compared to volume of low opacity. The Hosmer–Lemeshow goodness of fit between the measured and predicted values showed that volume of low opacity (*p* = 0.056) was higher than volume of high opacity (*p* = 0.022) and overall opacity (*p* = 0.021).

## 4. Discussion

In this single-center, retrospective study, we used collected data to investigate the correlation between the opacity volume of patients with COVID-19 pneumonia on CT image before treatment and the subsequent increase in oxygen demand.

In the comparison of the increased oxygen demand group and the nonincreased oxygen demand group, the median time from onset to CT was 3.5 days shorter for the increased oxygen demand group, whereas the opacity volume was 4.4 times larger despite early shooting. Individual differences may exist in the degree of symptoms that trigger a visit to the hospital and subsequent CT imaging, although CT imaging is often performed earlier in severe cases. This suggests that the early onset of symptoms due to the rapid spread of pneumonia after infection promoted early visits. Compared with high opacity volume, low opacity volume was considerably different between the increased oxygen demand group and the nonincreased oxygen demand group during the whole study period. The rapidly increasing GGOs may be associated with early-stage symptoms. Rorat M et al. retrospectively analyzed 61 patients with COVID-19 who underwent a CT scan due to suspicious symptoms of pneumonia during deterioration of health [23]. Quantitative CT was performed using deep learning and revealed a significantly higher severity of changes in type of GGOs and consolidation in patients with severe disease than in those with nonsevere disease. Although the results of this study tend to be the same as ours, the opacity of this cohort is much greater than ours. The possible causes are difference in the median time from onset to CT (12 days, compared to 9 days in our study) and that many patients may have poor respiratory status due to the strict definition of severe illness (room air oxygen saturation < 90%).

We also showed that volume of high opacity, low opacity, and overall opacity all proved to be significant variables and the risk of increases in oxygen demand increased as the volume of opacity became higher. In the Hosmer–Lemeshow goodness-of-fit test, the best fit between measured and predicted values was found for low opacity volume. Since the CT findings of pneumonia in COVID-19 change from low to high opacity as the disease worsens, measuring the volume of low opacity may be appropriate for predicting increases in oxygen demand.

In our subgroup study, the period was divided into three groups every 5 days, taking into consideration the ease of use when applied in actual clinical practice. There are subtle differences in previous studies regarding how to divide the cases into subgroups. Pan et al. [10] classified multiple patients with COVID-19 into four stages based on the quartiles of patients and degree of lung involvement. In the study by Wang et al. [8], numerous patients underwent CT several times. The time period was divided every 6 days as the median scan-to-scan interval was 6 days. In our cohort, the opacity volume peaked during the middle period. This time course was similar to that of previous studies [8,10,11]. Even if there were small differences in the grouping method, each study demonstrated a similar feature in the progression of pneumonia.

No significant difference was observed in the opacity volumes during the early period between the two groups, and the AUC for the increased oxygen demand was low. Thus, exacerbation could not be predicted by CT at the initial stage.

The AUC of the low opacity volume during the middle to late period exhibits moderate accuracy. The low opacity volume was significantly larger in the increased oxygen demand group during the middle period. It was still larger (eight times larger than that of the nonincreased oxygen demand group) during the late period but not significantly (*p* = 0.056). Conversely, the high opacity volume significantly increased during the late period in the increased oxygen demand group, whereas it decreased in the nonincreased oxygen demand group. The end AUC increased and exhibited moderate accuracy. Based on these data, low opacity is associated with exacerbation of the respiratory function after 6 days of onset, whereas exacerbation after 11 days might be correlated with the high opacity volume. The cause of the exacerbation of respiratory illness may be the appearance of new lesions due to the inability to control the illness or the nonreduction of existing GGOs/consolidation.

In this study, deep learning was adopted to automatically extract and quantify lung lesions from the CT scans of patients with COVID-19. Deep learning provides an efficient method for the detection and segmentation of the affected area on CT images. There are several papers that suggest the usefulness of CT evaluation using deep learning of COVID-19, but each has different end points and analysis methods [24,25]. Additionally, most of the studies focus on detection and diagnosis, and those that consider severity and prognosis are relatively rare [23]. The advantages of our method are as follows: 1. Predictions are based on a single CT, not a comparison of multiple CTs taken during the course of illness. Liu et al. conducted the first cohort study to predict outcomes in patients with COVID-19 using quantitative CT measurements, and they detected that the change of the CT image from day 0 to day 4 predicts progression to severe illness [24]. To safely perform CT for patients with COVID-19, a lot of human resources and occupancy of the CT room are required. Therefore, it is not recommended to perform multiple CTs, and it is highly evaluated to predict with a single CT.; 2. Consideration of the time from onset to imaging: Although there are several papers examining the predictive ability of quantitative analysis, it is difficult to say that it can contribute to the proper allocation of patients at an early stage due to the variation in CT scan timing.; 3. It focuses on quantitative data on pneumonia area and is easy to implement clinically, as it does not require an overly complex program. To the best of our knowledge, there are no studies that deal with a large number of early CTs in severe COVID-19 cases or objectively assess the predictive value of opacity, particularly in Japan.

Predictive techniques are required to prevent the spread of COVID-19 infection and effectively utilize limited medical resources. Due to the relative availability of RT-PCR in numerous hospitals and clinics, major interest has been directed toward predicting aggravation and early intervention. It is important to distribute severe patients to the appropriate facilities. As CT has become widespread in Japan, CT scans can be performed at small hospitals in the city. If a CT can predict the exacerbation of COVID-19 pneumonia, risk-aware treatment will be possible, and transfers for specialized treatment will be smoothly managed at the appropriate time.

This study has several limitations. First, the number of cases is small, particularly for a subgroup analysis, and the number of each group became smaller. Second, the details of CT imaging protocol and acquisition parameters at other hospitals are uncertain. Third, it is a retrospective, single-center study and it may not reflect the current or future situations for all societies because our hospital mainly dealt with patients with COVID-19 who had moderate-to-severe symptoms.

## 5. Conclusions

Deep learning-based quantitative analysis of the affected lung volume in the initial CT scans of patients with COVID-19 offers a predictive factor for determining the potential deterioration of the patient’s respiratory condition, which can guide treatment management and optimization of limited resources during the high demand of an outbreak.

## Figures and Tables

**Table 1:** Demographic and clinical characteristics of the study population.

Variables		All	Increased Oxygen Demand	Nonincreased Oxygen Demand	*p*-Value
**Number of subjects, n**		79	45	34	-
***Baseline characteristics***					
Age, years		62 [46, 77]	65 [54, 77]	51.5 [36, 71.5]	**0.025**
Sex, male, %		56 (70.9%)	38 (84.4%)	18 (52.9%)	**0.005**
Height, cm		168.5 [163.0, 177.8]	173.0 [165.0, 179.0]	164.4 [158.5, 169.3]	**0.008**
Weight, kg		65.0 [49.0, 74.9]	67.0 [55.0, 80.0]	56.5 [48.0, 70.0]	0.052
Body mass index		23.0 [20.8, 25.5]	24.4 [21.6, 28.1]	21.4 [19.1, 23.7]	**0.005**
Onset to CT duration, days		9.0 [6.0, 13.0]	7.0 [5.0, 9.0]	10.5 [8.3, 14.0]	**0.005**
***Status at the CT scan***					
Oxygen demand, %	none	56 (70.9%)	26 (57.8%)	30 (88.2%)	0.173
	exist	14 (12.7%)	10 (22.2%)	4 (11.8%)	
	no data	9 (11.4%)	9 (20.0%)	0 (0.0%)	
Mechanical ventilation, %		1 (1.3%)	1 (2.2%)	0 (0.0%)	1
Body temperature, °C		37.0 [36.7, 37.9]	37.6 [36.9, 38.0]	36.7 [36.5, 37.1]	**0.001**
WBC, 10^3^/μL		5500 [4025, 6900]	5300 [3670, 6750]	5600 [4550, 7250]	0.313
LYM, %		19.00 [10.60, 25.30]	15.65 [9.28, 19.67]	24.20 [18.15, 28.45]	**0.004**
CRP, mg/dL		4.96 [1.47, 11.97]	9.92 [3.76, 15.59]	2.13 [0.08, 7.88]	**<0.001**
LDH, U/L		292.0 [219.0, 409.0]	321.0 [284.0, 467.5]	242.0 [191.5, 314.5]	**0.001**
Lung volume, mL		4197.2 [3424.6, 4895.8]	4517.6 [3541.0, 5167.2]	3828.0 [3352.1, 4575.8]	0.059
Volume of opacity, mL		446.3 [63.8, 767.0]	585.3 [212.8, 916.1]	132.8 [10.7, 456.1]	**<0.001**
Volume of high opacity, mL		55.2 [8.2, 102.7]	71.4 [27.0, 165.4]	25.1 [0.9, 78.5]	**0.006**
Volume of low opacity, mL		336.2 [59.0, 655.1]	440.3 [165.6, 718.0]	95.1 [10.4, 405.6]	**0.001**

Continuous variables are expressed as median and interquartile range in brackets and were compared between the two groups using the Mann–Whitney U test. Categorical variables are expressed as numbers (%) and were compared between the groups using the *χ*^2^ test. Computed tomography, CT; white blood cell count, WBC; lymphocyte, LMY; C-reactive protein, CRP; lactate dehydrogenase, LDH; extracorporeal membrane oxygenation, ECMO.

**Table 2:** Volume of opacities in increased oxygen demand group and nonincreased oxygen demand group stratified by time from onset to CT scan.

Onset to CT Duration, Days	Type of Opacity	All	Increased Oxygen Demand	Nonincreased Oxygen Demand	*p*-Value
0–5 days	high opacity	15.3 [6.0, 71.4]	11.0 [5.7, 87.4]	16.2 [11.1, 17.9]	0.752
	low opacity	72.3 [33.8, 500.2]	239.5 [37.1, 508.9]	72.33 [33.8, 76.2]	0.343
	overall	87.3 [45.2, 526.8]	282.2 [42.4, 612.4]	87.3 [50.0, 96.2]	0.399
6–10 days	high opacity	57.6 [32.7, 126.8]	57.62 [42.5, 146.9]	65.2 [13.4, 87.3]	0.224
	low opacity	480.5 [286.7, 782.44]	551.8 [361.5, 1106.3]	330.7 [125.7, 527.7]	**0.044**
	overall	562.5 [329.0, 930.1]	607.6 [416.3, 1245.5]	430.98 [134.6, 613.2]	**0.040**
≥11 days	high opacity	57.8 [0.9, 127.6]	129.7 [46.0, 294.5]	5.28 [0.0, 62.1]	**0.018**
	low opacity	165.6 [10.0, 519.7]	425.1 [152.9, 648.6]	52.2 [1.5, 388.6]	0.056
	overall	325.0 [10.6, 749.7]	636.3 [252.9, 890.1]	55.73 [1.6, 446.3]	**0.027**

Abbreviations: computed tomography, CT.

**Table 3:** AUC of each type of opacity volume as a predictor of oxygen demand in each period.

Period	Number	Opacity	High Opacity	Low Opacity
Total	79	0.737 (0.624–0.851)	0.691 (0.572–0.811)	0.722 (0.607–0.837)
0–5 days	17	0.633 (0.342–0.925)	0.45 (0.132–0.768)	0.650 (0.362–0.938)
6–10 days	35	0.714 (0.529–0.898)	0.373 (0.167–0.579)	0.710 (0.523–0.898)
≥11 days	27	0.759 (0.574–0.943)	0.776 (0.593–0.96)	0.724 (0.528–0.919)

## Data Availability

All available data are presented within the article or are available on request from the corresponding author.
